# Predicting the distribution of *Phortica variegata* and potential for *Thelazia callipaeda* transmission in Europe and the United Kingdom

**DOI:** 10.1186/s13071-018-2842-4

**Published:** 2018-04-27

**Authors:** Jennifer Palfreyman, John Graham-Brown, Cyril Caminade, Paul Gilmore, Domenico Otranto, Diana J. L. Williams

**Affiliations:** 1 Institute of Veterinary Science, University of Liverpool, Liverpool, UK; 2 Infection Biology, Institute of Infection and Global Health, University of Liverpool, Liverpool, UK; 3 Epidemiology and Population Health, Institute of Infection and Global Health, University of Liverpool, Liverpool, UK; 4 NIHR, Health Protection Research Unit in Emerging and Zoonotic Infections, Liverpool, UK; 5 Liverpool Veterinary Parasitology Diagnostics, University of Liverpool, Liverpool, UK; 6 Dipartimento di Medicina Veterinaria, University of Bari, Bari, Italy

**Keywords:** *Phortica variegata*, *Thelazia callipaeda*, Dipteran vectors, Zoonosis, Ecological niche modelling

## Abstract

**Background:**

Male fruitflies *Phortica variegata* (Drosophilidae, Steganinae) are the intermediate host of the zoonotic nematode *Thelazia callipaeda* (Spirurida, Thelaziidae). More than 10 years ago, when *T. callipaeda* was confined to remote regions of southern Italy, ecological niche models were used to predict the potential distribution of *P. variegata* across Europe and the likely risk of the nematode spreading through infected dogs travelling to/from endemic regions. As predicted, over the last 10 years *T. callipaeda* has spread rapidly across Europe. Recently, we identified the potential for its introduction to the UK through infected dogs travelling to/from endemic regions of mainland Europe.

**Methods:**

Here updated information is used to re-evaluate the model-predicted European, and specifically, UK distribution to determine the likelihood of *T. callipaeda* becoming established. Additionally, the UK distribution of *P. variegata* was further investigated through snapshot fly trapping at model-predicted locations.

**Results:**

Ecological niche modelling using Genetic Algorithm for Rule-set Prediction (GARP) analysis suggests a European range similar to that described previously, with some indication of potential spread further eastward. Finer scale UK mapping suggested that *P. variegata* presence was limited mostly to southern England, but highlighted regions where *P. variegata* has not been documented previously. The arbitrary fly trapping identified activity of *P. variegata* at two locations where the species has been found previously late in the season. No specimens were collected at model-predicted locations, although habitat suitable for the species was identified.

**Conclusions:**

GARP-model prediction of *P. variegata* distribution suggests presence of suitable conditions in previously undocumented regions of the UK and Europe and highlight the possibility for further spread of *T. callipaeda* across Europe, including the UK. Further work to validate the *P. variegata* UK model with field data will help improve its accuracy in predicting suitable areas, whilst surveillance of sylvatic definitive host species in such locations is advised to monitor for evidence of autochthonous *T. callipaeda* transmission.

## Background

*Thelazia callipaeda* (Spirurida: Thelaziidae) is a vector borne, zoonotic nematode of animal and public health importance capable of infecting a range of host species, including dogs and cats, foxes, wolves, rabbits and humans [1]. Adult worms reside in the conjunctiva and surrounding structures of their definitive host, causing a range of clinical outcomes from asymptomatic carriage through to severe ocular pathology including blepharitis, conjunctivitis and corneal ulceration [2]. In recent years, *T. callipaeda* appears to have spread rapidly through Europe: since 2003 the list of endemic countries has expanded from Italy [3], where autochthonous transmission was first recorded in Europe, to include France, Switzerland, Germany, Spain, Portugal, Bosnia and Herzegovina, Croatia, Romania, Bulgaria, Hungary, Greece, Slovakia and Serbia [4–15]. Zoonotic infections have been diagnosed in endemic regions of Europe on several occasions, demonstrating the importance of its spread to public health [2, 15–17]. To date, DNA sequence analysis of the mitochondrial cytochrome *c* oxidase subunit 1 (*cox*1) gene performed on specimens collected from several different host species across Europe has demonstrated presence of a single haplotype (h1). This suggests spread of *T. callipaeda* in Europe has occurred from a single introduction event and/or that this haplotype (h1) is well adapted for survival and transmission in Europe [18].

Travel of companion animals between European countries provides an opportunity for further dissemination of *T. callipaeda*. For example, in 2016 patent infections were imported to the UK on at least two occasions in animals with a history of travel to *T. callipaeda* endemic areas [19], whilst the introduction of the parasite to Spain is thought to have occurred through the importation of infected hunting dogs from Italy and France [8]. Furthermore, zoonotic infections acquired during travel abroad may also facilitate the introduction of *T. callipaeda* to non-endemic countries on a global scale, emphasising the need for a “One Health” approach for the surveillance of such disease [20].

A critical determinant of *T. callipaeda* spread and distribution is the presence of intermediate host species. In Europe, males of *Phortica variegata* (Drosophilidae: Steganinae) are intermediate hosts and vectors under natural conditions [21]. These flies are typically associated with oak (*Quercus* spp.) woodland habitats and fruit farms [21] and primarily feed on fermenting sap runs and fruit. Additionally (and importantly), males exhibit zoophagic behaviour, feeding on the lacrimal secretions of humans and animals [21]. It is through this behaviour that male flies transmit *T. callipaeda*, becoming infected through the ingestion of L1 larvae when feeding on infected hosts, and depositing infective L3 larvae onto the eyes of new definitive hosts when they feed again. Development from L1 to L3 occurs on the surface of testes of the fly, at which point infective L3 larvae migrate to the oesophagus and proboscis to be transmitted to a definitive host [22]. This process typically takes 14–21 days from the point of ingestion. In southern Europe, flies are active from May to October with optimal temperatures (20–25 °C) and humidity (50–70%) present in July and August leading to greater activity and likelihood for *T. callipaeda* transmission at this time [21, 23]. Ecological niche modelling in 2006 demonstrated that the potential distribution of *P. variegata* ranged widely across southern and central Europe, extending as far north as Denmark, the southern tip of Sweden and south-eastern England [23].

Whilst *P. variegata* is present in the UK, it is currently listed as a species of conservation concern under Section 41 of the Natural Environment and Rural Communities Act 2006 [24]. There have, however, been a number of observations of this species in recent years [25]. Additionally, questions exist concerning its biology and ecology in the UK including the length of its active flying season. Understanding both geographical distribution and the length of flying season of *P. variegata* are of importance when evaluating the potential for *T. callipaeda* introduction to the UK and the likelihood of autochthonous transmission occurring, especially in wild carnivores such as foxes.

The aim of this study was to re-visit the Genetic Algorithm for Rule-set Prediction (GARP) model analysis described by Otranto et al. [23] to predict ecological niche distribution for *P. variegata* across Europe using updated climate data and *P. variegata* records. This is important given recent climate change trends in Europe and the UK. Furthermore, to help determine whether autochthonous transmission of *T. callipaeda* might occur, particular consideration was given to the UK through fine-scale mapping and field sampling for model validation.

## Methods

### Ecological niche modelling

GPS co-ordinates were obtained from European records [26, 27], and derived from the UK literature [25, 28–30]. To avoid retrospective extrapolation of climate data, only locations recorded post 1950 were selected for inclusion. Only a single observation per GPS coordinate was included to avoid duplication of data. These selection criteria yielded 243 locations for *P. variegata* from across Europe (at longitudes from 8°W - 48°E and latitudes from 36°N - 63°N) including 19 from the UK. Seventy-one locations were newly identified since 2006.

The software application DesktopGarp (http://nhm.ku.edu/desktopgarp) was used to model *P. variegata* distribution across Europe and, specifically, the UK. For European models, spatial datasets for fixed variables included mapped altitude (m) and slope from the ETOPO1 dataset [31], meteorological records (1959–2015) for annual mean rainfall (mm), temperature (°C), diurnal temperature range or DTR (°C), wet days (defined as days above 1 mm rainfall), potential evapotranspiration or PET (mm) [32], and solar irradiance (W/m^2^) derived from satellite estimates [33]. All fields were interpolated to a regular 0.25° × 0.25° spatial grid (at longitudes from 23.875°W - 45.375°E and latitudes from 30°N - 71.375°N).

For UK models a higher spatial resolution was obtained through interpolation to a regular 0.05° × 0.05° spatial grid (at longitudes from 12°W - 4°E and latitudes from 49°N - 60°N). Altitude data was again derived from the ETOPO1 dataset, whilst meteorological records (1977–2015) for temperature, DTR, wet days, sunshine duration (hours per day) and PET data were based on the UKCP09 gridded observation dataset [34] available at 5 × 5 km.

**Fig. 1:**
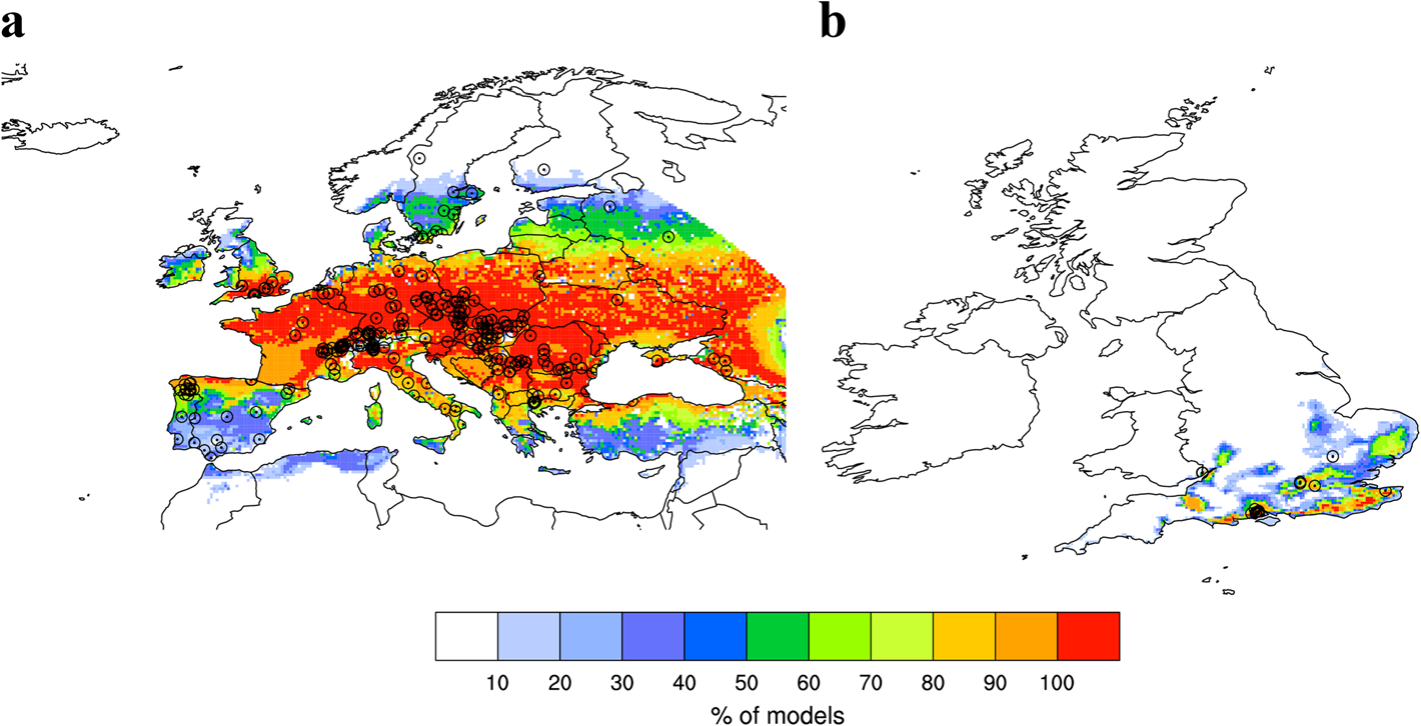
Predicted geographical distribution of *Phortica variegata* across (**a**) Europe (0.25° × 0.25° spatial grid, 23.875°W - 45.375°E and 30°N - 71.375°N) and (**b**) the UK (0.05° × 0.05° spatial grid, 12°W - 4°E and 49°N - 60°N). Heat map coloration denotes the percentage of models (*n* = 16 and *n* = 33, respectively) agreeing on suitability for each grid point. Observed locations of *P. variegata* used to train the models are depicted by location points

The model for Europe was calculated using all 243 points of *P. variegata* presence dating from 1959–2015. For the UK, 19 points were available dating from 1977–2015. As part of the GARP modelling process, pseudo-absence data points equal to the number of presence points were also randomly generated and incorporated into the analyses. The reader is invited to refer to Stockwell and Peter, 1999 for further details about this algorithm [35]. Statistical parameters set for both models were 100 runs, 0.01 coverage and 1000 of maximum iterations with rules set as negated range and logistic regression only. Models with a non-zero omission error and significance values of lower than 0.05 (95% confidence interval) were retained. This resulted in 16/100 and 33/100 models being used for Europe and the UK, respectively. Heat maps were constructed showing the percentage of models agreeing on suitable areas for *P. variegata* for each interpolated spatial grid point (see Fig. 1), instead of using a median estimate based on the multi-model ensemble [23].

### Field sampling

To evaluate model accuracy, and to try and identify and generate additional *P. variegata* occurrence data, six locations were visited in late summer 2017. Firstly, to establish an effective sampling protocol three locations where *P. variegata* has previously been identified were visited in the last week of August (Table 1). Following successful identification of *P. variegata* at two of these locations, a further three locations were sampled the following week (Table 1). These locations were areas of dense deciduous woodland (including *Quercus* spp.) located at (or close to) areas where the newly developed UK GARP model predicted the potential presence of *P. variegata*. This was achieved by overlaying the fine scale (0.05° × 0.05° spatial grid) UK map in Google Earth Pro® (https://www.google.com/earth/) [36].

**Table 1:** Baiting locations and observations from field sampling

Bait site	Latitude	Longitude	Date	Reference	*Phortica* presence (Notes)
1. Ban-y-gor, Gloustershire	51.6678° N	2.6703° W	28/08/17	[30]	No *Phortica* collected. (Deciduous woodland containing *Quercus* spp. Baited oak with small drying sap run. Overcast day with cool ambient temperatures).
2. New Forest, Hampshire	50.8421° N	1.5147° W	29/08/17	[25]	Three *Phortica* (1M, 2F) collected by sweep net. (Oak woodland. Sunny day with warm ambient temperature).
3. High Standing Hill, Windsor Forest	51.4694° N	0.6714° W	30/08/17	[28]	One *Phortica* (F) collected by sweep net. (Deciduous woodland containing *Quercus* spp. Initially warm sunny day with conditions deteriorating to rainy and overcast with cool ambient temperatures).
4. Collin’s Wood Nature Reserve, Gloucestershire	51.9477° N	2.3682° W	04/09/17	Model-predicted^a^	No *Phortica* collected. (Suitable habitat identified: deciduous woodland containing *Quercus* spp. and commercial apple orchard adjacent. Baited oak with large fragrant sap run. Overcast day with warm ambient temperatures and humid conditions).
5. Castle Neroche, Somerset	50.9360° N	3.0342° W	05/09/17	Model-predicted^a^	No *Phortica* collected. (Suitable habitat identified: Oak forest. Wet and windy conditions with cool ambient temperatures).
6. Rendlesham Forest, Suffolk	52.0799° N	1.4329° E	06/09/17	Model-predicted^a^	No *Phortica* collected. (Suitable habitat identified: Oak forest. Sunny day with warm ambient temperatures).

^a^Model-predicted locations were identified by overlaying GARP model-generated fine-scale map of UK *P. variegata* distribution (Fig. 1b) on satellite images to locate areas of suitable habitat (woodland) in regions with high model-predicted likelihood of *P. variegata* presence

Locations with areas of established oak woodland with scrub undergrowth were chosen for trapping due to their suitability as habitat for *P. variegata*. Sampling at each location consisted a four hour period between 10:00 and 14:00 h on the date specified (Table 1). Bait consisted of fermented fruit (chopped banana, apple and pear sealed in an airtight container and left at room temperature for 48 h) used with either a sweep net to catch visiting flies, or in four bottle traps prepared as previously described [21, 37] and hung in the vicinity for the duration of the sampling period. Both bait and bottle traps were hung at a height of 1–2m above ground level.

All the specimens collected were preserved in 70% ethanol on the day of trapping to retain both morphological and molecular integrity for species identification, dissection and (if indicated) PCR analysis. *Phortica variegata* were positively identified through comparison to previously described morphological features [23]. Male *P. variegata* specimens were dissected to check for *T. callipaeda* infection status: the thorax (oesophagus), head and proboscis were dissected and examined under low power magnification (×4–10) for infective third stage larvae, whilst testicular tissue was removed and examined under higher magnification (×20–40) for encysted first and second stage larvae. Had nematodes been observed, the intention was to establish their identity through a combination of morphological description and molecular analysis.

## Results

The European distribution predicted by the GARP model (Fig. 1a) indicated that large areas are suitable for the development of *P. variegata*, with the geographic range extended further east (e.g. over Ukraine, Russia and northern Turkey) compared to that published by Otranto et al. [23]. Finer scale mapping of *P. variegata* distribution in the UK (Fig. 1b) showed it was likely to be limited to the south and east of England. This included locations where *P. variegata* has been identified previously (south Gloucestershire, Dorset, Hampshire and Kent), and also regions where no prior records exist including Surrey, Sussex, Suffolk, Essex, Somerset and Worcestershire (Fig. 1b). This map has been made publicly accessible through the University of Liverpool’s online research data catalogue (http://datacat.liverpool.ac.uk/id/eprint/434) [36].

**Fig. 2:**
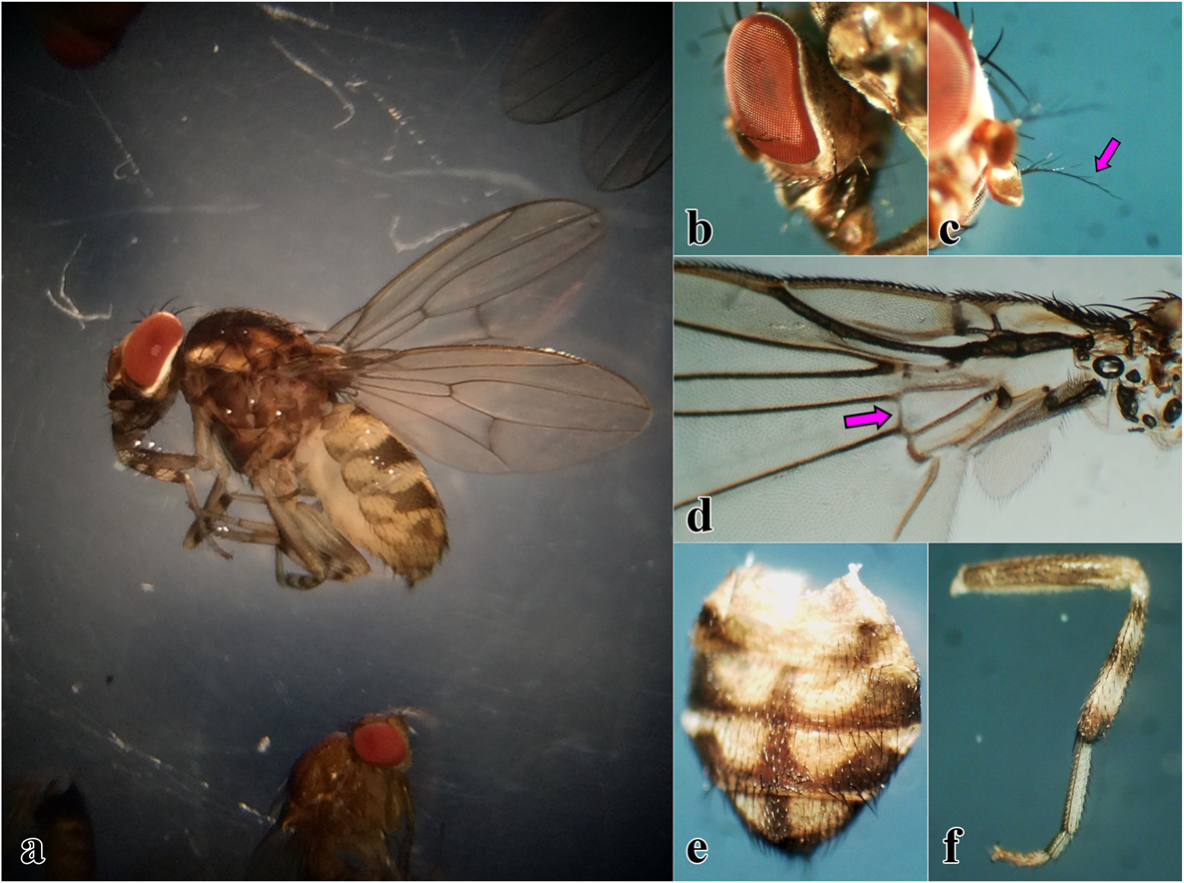
Morphological features of *Phortica variegata* including (**a**) an intact female; (**b**) red coloured eyes encircled by a pale white ring; (**c**) the arista of the antenna with four short dorsal branches (arrow) decreasing in length towards the apical tip; (**d**) an additional cross-vein separating the discal and second basal wing cells (arrow) plus two interruptions of the costal vein- a characteristic feature of Drosophilidae; (**e**) the abdomen with three transversal and one longitudinal dark band on a paler sub-colour; and (**f**) legs with three dark bands around the tibia and dark coloured coxae and femur

Field sampling at locations where *P. variegata* had previously been identified yielded four specimens in two of the three localities (Table 1). All *P. variegata* specimens were collected through sweep net trapping around fermented fruit bait, none were caught in the bottle traps. Specimens were identified morphologically as *P. variegata* based on key features (Fig. 2): briefly, specimens measured 3.5–5 mm in length, with red coloured eyes encircled by a pale white ring (Fig. 2b); the arista of each antenna had three to six short dorsal branches of decreasing length towards the tip (Fig. 2c); the scutum was patterned with dark, confluent spots; the discal and second basal wing cells were separated by an additional cross-vein (Fig. 2d); the abdomen had a yellow and brown pattern consisting of three transversal and one longitudinal dark band on a paler sub-colour (Fig. 2e); the legs had three distinctive dark bands around the tibia, dark coloured coxae and femur, with the latter also having a paler base and apex (Fig. 2f) [23].

No *P. variegata* were collected from the three locations predicted to be suitable by the model, although at all three suitable habitat was found in the form of oak (*Quercus* spp.) woodland with underlying scrubland (Table 1). Furthermore, evidence of active sap runs were detected on some trees and at one location a commercial apple orchard was also found in the immediate vicinity (Table 1). All six locations had public footpaths. Owners were regularly observed walking their dogs through these areas. A single male *P. variegata* was caught at one location where *P. variegata* had been documented previously (Table 1), but no nematode larvae were detected following dissection.

## Discussion

Our study updates the ecological niche model by Otranto et al. [23], which was based on 240 known locations of *P. variegata* from across Europe and used climatic data from 1960–1990. Subsequent reports of autochthonous transmission in multiple European countries in the intervening period have proven the validity of that approach, namely determining the ecological and environmental conditions suitable for *P. variegata* [5–9, 11–15]. In the current study, ecological niche model analysis using up to date climate information and 71 new *P. variegata* locations indicates the potential for further introduction of *T. callipaeda* to new regions of Europe, including the UK.

The ecological niche models presented here for Europe are largely in agreement with those described previously [23], but suggest an increase in the distribution range of *P. variegata* eastward to include new regions of the Ukraine, Turkey and Russia. To date, autochthonous transmission of *T. callipaeda* in Europe has been limited to a single haplotype (h1), in contrast there are at least twenty additional haplotypes present in southeast Asia [18, 38, 39]. Recent phylogenetic analysis of *T. callipaeda* isolates identified a divergence between European h1 and those haplotypes present in Asian countries during the Pleistocene epoch, suggesting two separate sub-populations [39]. The presence of just a single haplotype in Europe may be the result of either a specific genetic adaption of *T. callipaeda* h1 which enables its survival and propagation in Europe, or an indication that its spread in Europe was the result of a single introduction of *T. callipaeda* h1 to the continent. If the latter is true, expansion of *P. variegata* range eastward increases the likelihood of further introductions of *T. callipaeda* from Asia, including new haplotypes with unknown consequences for the biology and epidemiology of *T. callipaeda* in Europe, specifically its prevalence, incidence and zoonotic potential.

GARP model analysis suggests the most suitable areas for *P. variegata* in the UK are in southeast England. In many instances these regions intersect with national parks such as the New Forest, Windsor Forest, Wye Valley, South Downs and parts of the Kent Downs; many of these locations are popular areas for dog walking. This scenario could bring competent definitive and intermediate hosts of *T. callipaeda* into close proximity. If dogs infected with *T. callipaeda* as a result of travel to endemic European regions are exercised in such areas, autochthonous transmission to *P. variegata* in the UK is a possibility. Furthermore, such national parks are home to numerous sylvatic species (e.g. red foxes, hares and rabbits) which are themselves competent definitive wild hosts for *T. callipaeda* [1, 40]. This readily available wildlife reservoir further increases the possibility of *T. callipaeda* becoming established in the UK.

The single male *P. variegata* specimen collected was not infected with *T. callipaeda*. However, given that the infection rate in *P. variegata* is ~1% under natural conditions [21] the probability of detecting autochthonous transmission by this method is very low. For surveillance purposes, a more realistic approach would be to passively monitor *T. callipaeda* infection in sylvatic species in regions where *P. variegata* presence is already known.

Overlaying the UK map as a layer in Google Earth Pro® allowed the identification of potential *P. variegata* sites by locating areas of woodland within these hotspot areas [36]. This approach demonstrates a major advantage of ecological niche modelling, since it facilitates a more targeted approach to both field sampling for *P. variegata* and surveillance for autochthonous *T. callipaeda* transmission. Whilst we were unable to confirm *P. variegata* presence in locations predicted by the new GARP model, we were able to identify habitat suitable for the species, specifically extensive *Quercus* spp. woodland. The inclement weather conditions (Table 1) and relative lateness in the season for sampling may have hindered our detection of *P. variegata* even if they were present. Further field sampling at such sites in summer months would help establish the presence or absence of *P. variegata* more definitively. Additional observations could then be incorporated into updated versions of the UK GARP model for *P. variegata* distribution to improve its accuracy, or derive risk maps on a per month basis.

The vector competence of *P. variegata* from geographical areas where *T. callipaeda* is not yet present, such as the USA, has been recently demonstrated under laboratory conditions [41], confirming the potential for autochthonous transmission in areas where the parasite is not endemic but the vector occurs, such as the UK. We have confirmed that, under favourable climatic conditions, adult *P. variegata* were still active in the UK in the last week of August. Falk [25] noted previously that *P. variegata* activity in the UK was observed in June and July, but that the full extent of their active flying season was unknown. Current evidence would suggest an active flying season in the south of the UK from May through to September [25, 30]. Importantly, with respect to its role as a vector of *T. callipaeda*, this would enable transmission in the UK to occur within a single flying season, since the minimum time required for development of first-stage larvae to infective third-stage larvae is 14–21 days [22].

The ability of infected *P. variegata* to successfully overwinter has been demonstrated experimentally [22]. Additionally, *T. callipaeda* L3 and L4 larvae recovered from *P. variegata* and dogs, respectively, at the beginning of the active flying season suggesting delayed transmission to the following flying year is possible under natural conditions [21]. In cooler northern latitudes the ability of infected *P. variegata* to overwinter may prove to be an important additional feature in determining whether such fly populations are capable of sustaining autochthonous transmission of *T. callipaeda*.

## Conclusions

By updating and developing ecological niche models of *P. variegata* distribution across Europe and the UK, respectively, we have confirmed its predicted range and indicate the potential for expansion to new regions. In the UK, this has implications with respect to the potential for autochthonous transmission of *T. callipaeda* following its introduction from mainland Europe. Passive surveillance of sylvatic species is suggested to monitor for autochthonous *T. callipaeda* transmission. In addition, research to determine and improve the accuracy of GARP models and/or introduce temporal components to consider seasonal and annual range variation would further help monitor the distribution of *P. variegata* and the subsequent risk of *T. callipaeda* infection to both human and animal populations.

## Abbreviations

GARP: Genetic Algorithm for Rule-set Prediction
JNCC: Joint Nature Conservation Committee
NIHR: National Institute for Health Research
UK: United Kingdom (of Great Britain and Northern Ireland)
USA: United States of America
